# A Worldwide Bibliometric Analysis of Published Literature Assessing Fear of COVID-19

**DOI:** 10.3390/clinpract14030054

**Published:** 2024-04-23

**Authors:** Jesús Cebrino, Silvia Portero de la Cruz

**Affiliations:** 1 Department of Preventive Medicine and Public Health, Faculty of Medicine, University of Seville, Avda. Sánchez Pizjuán, S/N, 41009 Seville, Spain; jcebrino@us.es; 2 Department of Nursing, Pharmacology and Physiotherapy, Faculty of Medicine and Nursing, University of Córdoba, Avda. Menéndez Pidal, S/N, 14071 Córdoba, Spain; 3 Research Group GE10 Clinical and Epidemiological Research in Primary Care, Instituto Maimónides de Investigación Biomédica de Córdoba (IMIBIC), Hospital Universitario Reina Sofía, 14071 Córdoba, Spain

**Keywords:** bibliometric, COVID-19, fear, mental health, publications

## Abstract

Many people experience intense fear of COVID-19. The purpose of this study was to provide a comprehensive visual overview of the published literature from 2020 to 2022 assessing fear of COVID-19. From 2020 to 2022, we employed the Scopus database to conduct a bibliometric analysis. We used the VOSviewer program to perform the author co-citation analysis, Mapchart to produce a worldwide map, and Wordart to make a word cloud image. From the 1769 records examined, 1654 (93.50%) were articles, with English being the most common language (96.31%). From 2020 to 2022, annual citations experienced significant growth (R^2^ = 99.91%; *p* = 0.0195). The Institut National de la Santé et de la Recherche Médicale (INSERM, France) and China led in terms of publication output (n = 36; n = 255). M. D. Griffiths authored the highest number of articles (n = 21). The most active journal was the *International Journal of Environmental Research and Public Health* (n = 146), and the most prevalent keyword was “human/s” (11.51%). This bibliometric analysis evaluates the quality of the research on fear of the pandemic and the crisis management of COVID-19, which can provide managers and researchers with crucial insights for future decision making.

## 1. Introduction

The World Health Organization announced on 30 January 2020 that the emerging novel coronavirus, known as severe acute respiratory syndrome coronavirus-2 (SARS-CoV-2), constituted a global public health emergency [1]. Since then, the scientific community’s response to coronavirus disease 2019 (COVID-19) has resulted in an enormous amount of research that has moved at breakneck speed through the scientific publishing [2]. Nevertheless, the public was greatly affected by misleading and unfounded information while the COVID-19 pandemic was in its early phases; this was caused by inaccurate reporting and fundamental misunderstandings about COVID-19 [3,4]. For this reason, the pandemic led to widespread panic and predisposed people to mental health problems that originated from a fear of the significant risk of illness and death [5]. As there were no effective pharmacological interventions or vaccines for treating or avoiding COVID-19, the only viable strategies in response to the pandemic were public health measures, such as self-isolation, physical separation, and lockdown, which were the only effective ways to respond to the outbreak [6]. As the global prevalence spread, individuals began to hoard healthcare equipment, isolate themselves, limit interaction with others, and live in a perpetual cycle of health anxiety and fear of SARS-CoV-2 [7,8].

Fear is a negative psychological reaction triggered by the perception of danger and feelings of insecurity [9,10], and it is regarded as a functional, adaptive, and transient response to events that results in sudden physiological alterations [11]. In fact, because infections are transmissible, imminent, and invisible, they are a very effective source of fear [12]. Schimmenti et al. [13] proposed four components of fear of COVID-19: (i) fear of and for one’s own body, as an individual acts as both a possible vector and a victim; (ii) fear of and for other people, which is also connected to the stress caused by the obligatory social distancing from interpersonal relationships; (iii) fear of both ignorance and knowledge of the virus, as the information needed for protection can also be difficult and anxiety-producing; and (iv) fear of both personal action and inaction, as fear has behavioral consequences. Knipe et al. [14] examined Google search trends, revealing an excessive rise in fear and in the number of searches about self-care.

Several studies have explored the most common behavioral issues related to fear of COVID-19. Thus, a negative association between vaccine hesitancy and a moderate/high fear of personally contracting COVID-19 has been found [15]. On the other hand, fear does not always play an effective role with regard to increasing the intention to comply with preventive measures [16,17]. Higher levels of fear were associated with a lower level of COVID-19 preventive behaviors [17], contradictory measures [18], and a growth of public mistrust in governments and health and scientific institutions [19].

These fears should be screened correctly [20], through psychological interventions in some cases [21]. To do this, healthcare providers require a reliable tool to analyze an individual’s psychological reactions to COVID-19 throughout the pandemic [22]. In this regard, various authors created different psychometric instruments to gain greater insight into these psychological responses to fear of COVID-19 [23,24,25,26,27]. Since then, an increasing number of publications have highlighted fear of acquiring the illness as one of the most damaging psychological effects of the pandemic [28].

To address the increasing amounts of existing literature assessing fear of COVID-19, we conducted a bibliometric analysis in this research; this statistical method provides information about a particular field of study [29,30], deepening the relationships between articles, citations, co-citations, and keywords, as well as providing strong visualization, allowing readers to identify future research interests easily and clearly in the research area [31]. Bibliometric analysis can therefore be used to investigate the published literature assessing fear of COVID-19, and to the best of our knowledge, no such bibliometric studies have been conducted in this field so far. The purpose of this bibliometric analysis was to provide a comprehensive, visual overview of the published literature assessing fear of COVID-19 from 2020 to 2022.

## 2. Materials and Methods

### 2.1. Selection Strategy

**Figure 1:**
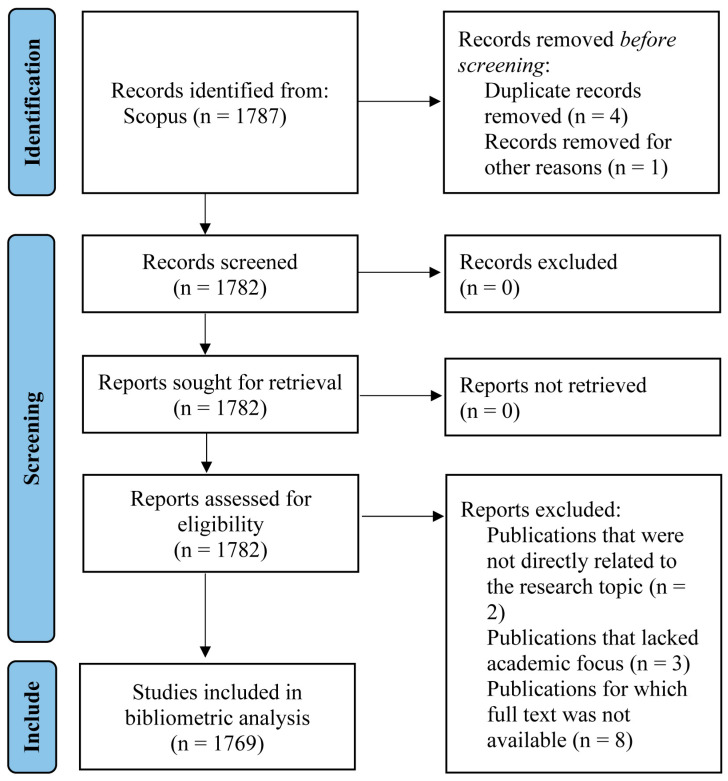
PRISMA flow diagram of the publication selection process.

A bibliometric analysis of the published literature in the Scopus database was performed from January 2020 to December 2022. The results of the literature searches and article selection process are illustrated in Figure 1; the Preferred Reporting Items for Systematic Reviews and Meta-Analysis (PRISMA) guidelines are followed [32].

The Scopus database was chosen because it is an extended, abstract citation database which covers journals and has smart tools for tracking, analyzing, and visualizing research from over 25,100 journals (most of which are peer-reviewed), 210,000 books, and 9.8 million conference papers [33].

Our searches focused on one main topic: the published literature assessing fear of COVID-19. The search formula, which is shown in Table S1 (Supplementary Materials), used the field code “TITLE-ABS-KEY”, and the Scopus database was searched using a set of search terms provided by the author; these terms included (a) “fear”, (b) “COVID-19”, and (c) “instruments assessing fear of COVID-19”. The synonyms of these keywords were linked using the Boolean operator “OR” and the Boolean operator “AND” to link queries (a), (b), and (c). In addition, all the included articles were examined manually to identify articles that were not relevant to the quantitative analyses or were not related to the main topic.

### 2.2. Selection Criteria

After removing any irrelevant published literature, the authors of the present study (JC and SPdlC) independently validated the data input and collection based on their expertise and perspectives in this area of research before repurposing the dataset for the bibliometric analyses. Any discrepancy between the two researchers was addressed and settled by agreement.

The search strategy was designed as a sensitive search, where the aim was to capture as many documents associated with the study topic as possible [34]. Therefore, the present bibliometric study included the following: (a) articles, reviews, letters, notes, editorials, short surveys, conference papers, book chapters, and books and (b) all languages. Next, the publications selected in the preceding stage were subjected to the exclusion criteria. Figure 1 depicts the publications that were removed, which included errata, duplicates, publications unrelated to the research topic, publications without an academic focus (i.e., lacking a clearly defined research focus or organized methodology), and documents for which the complete text was not accessible.

### 2.3. Data Extraction

To extract the data, we exported all the data to a CSV format to analyze the various bibliometric measures, including the author’s name and affiliation, the journal name, the article title, and the keywords.

### 2.4. Data Visualization and Statistical Analysis

VOSviewer software was used to perform the author co-citation analysis (version 1.6.18). This software allows us to visualize bibliometric maps and to analyze bibliographic and thematic linkage [35]. The co-citation analysis principle is a technique that quantifies relationships and connections between papers and determines how frequently two articles are cited by a third paper [36]. In this study, the object of co-citation analysis was the author, and a greater frequency of author co-citation therefore indicates a stronger link between them [37]. To avoid duplications, the authors’ names were standardized.

We generated a tagcrowd using the Wordart tool (https://wordart.com, accessed on 25 January 2023) and used the Mapchart website (https://mapchart.net/world.html, Mapchart website: accessed on 25 January 2023) to create the global map. The following terms: article(s), cross-sectional study(ies), major clinical study(ies), questionnaire(s), controlled study(ies), prevalence, survey(s), health survey(s), cohort analysis, and follow up were omitted because they were deemed unimportant to our research. All the figures were created using Microsoft^®^ Excel^®^ for Microsoft 365 MSO (version 2301, Microsoft Corp., Redmond, WA, USA).

Descriptive and inferential statistical analyses were conducted using frequencies of document types, subject categories, languages, number of annual publications and citations, journal names, authors’ names and affiliations, countries, article titles, and keywords. The number of citations per publication (CPP) was expressed according to the mean and the standard deviation. The statistical analysis was performed using the IBM SPSS Statistical software version 26.0.0 (IBM Corp, Armonk, NY, USA), licensed to the University of Seville (Spain).

## 3. Results

This study found a total of 1769 publications between 2020 and 2022.

### 3.1. Document Types, Subject Categories, and Languages of Publications

The vast majority of the documents (n = 1654; 93.50%) were articles. There were fewer than 100 reviews (n = 57; 3.22%) and letters (n = 45; 2.54%). Finally, conference papers (n = 5; 0.28%), editorials (n = 5; 0.28%), and notes (n = 3; 0.17%) accounted for fewer than 10 publications.

“Medicine” obtained by far the highest percentage among the subject categories (84.91%), followed by “Psychology” (11.48%), “Neuroscience” (10.29%), “Environmental science” (8.48%), and “Nursing” (5.43%) (Table 1).

**Figure 2:**
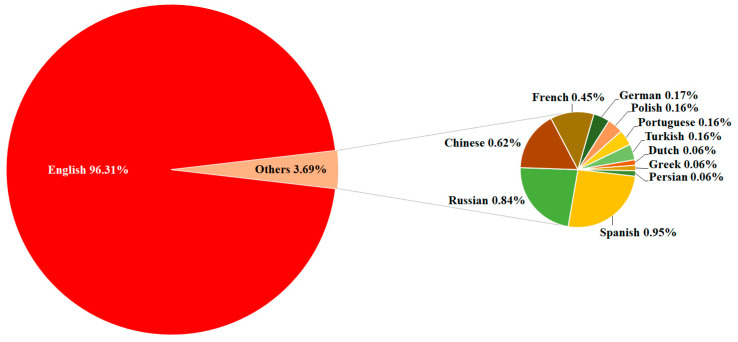
Language of publications assessing fear of COVID-19 (2020–2022).

The situation was clear in terms of the publication language (Figure 2), with English (97.29%) being the most common. The category “Others” showed the least commonly used languages in the study documents, with Spanish (0.96%), Russian (0.85%), Chinese (0.62%), and French (0.45%) being among the languages in this category.

### 3.2. Trends of Publications and Citations

In the period from 2020 to 2022, the number of annual documents was not significant year by year (R^2^ = 64.31%; *p* = 0.4076). Nevertheless, the trend in the number of annual citations increased exponentially, with an R^2^ coefficient of 99.91% (*p* = 0.0195).

### 3.3. Most Productive Journals

Table 2 shows the top 10 most active journals related to the published literature assessing fear of COVID-19 (2020–2022). The only journals with more than 100 documents were the *International Journal of Environmental Research and Public Health* (n = 146) and *Frontiers in Psychiatry* (n = 115). In this regard, the first journal is in the second quartile of the 2022 SCImago Journal Rankings. Otherwise, the top 10 journals were in the first or second quartiles and had high impact factor values. *Psychiatry Research*, on the other hand, was the journal with the highest number of annual citations (n = 4303), and it obtained the highest CPP (159.37).

### 3.4. Analysis of Authors

Table 3 shows the top 10 authors who mainly published articles related to assessing fear of COVID-19 (2020–2022). M. D. Griffiths had the most publications (21 records), followed by S. Chung (19 records), A. H. A. Pakpour (18 records), and C. Y. Ling (16 records). C. Pieh received the highest number of annual citations (n = 766) and obtained a CPP of 76.70.

**Figure 3:**
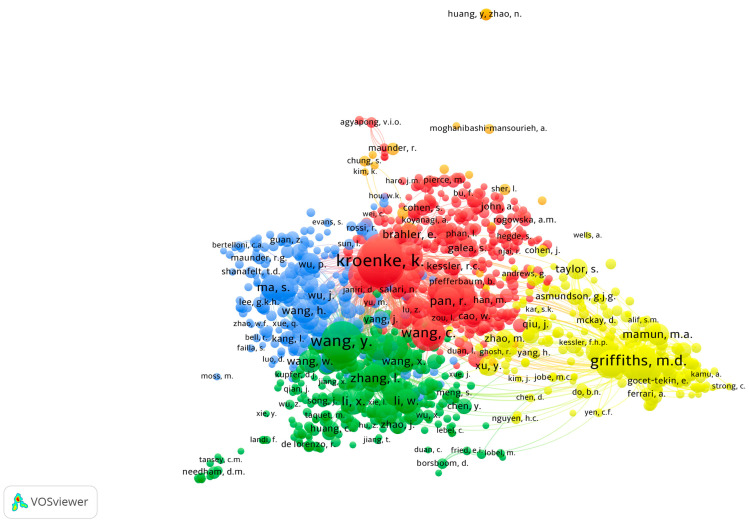
Author co-citation map related to published literature assessing fear of COVID-19 (2020–2022).

The author co-citation analysis is illustrated in Figure 3. The threshold of 20 citations per author was met by 1194 out of the 84,275 authors. Each node denotes an author, and the size of the node indicates how many times the researcher has appeared in publications. A co-citation relationship is indicated by a link between two nodes. Each link has a strength: the thicker the link, the stronger the relationship. The nodes are also grouped together according to similarity.

The author co-citation analysis depicted five distinct clusters, each representing a field of this research topic: blue (top left), green (bottom left), orange (top middle), red (middle), and yellow (right). The most cited authors in the blue cluster dealt mainly with mental health outcomes among health professionals. The most cited authors in the green cluster mainly studied mental health and clinical characteristics and the outcomes of hospitalized patients with COVID-19. The most cited authors in the orange cluster focused on mental health in different groups of the population or in the general population during the COVID-19 pandemic, or they focused on the measures used to assess the anxiety response to the viral pandemic during the pandemic. The most cited authors in the red cluster mainly produced publications about the prevalence and associated factors of anxiety symptoms and the use and validation of different surveys. Finally, the most cited authors in the yellow cluster dealt mainly with the psychometric properties of different fears of COVID-19 and with questionnaires and other related surveys and studies.

### 3.5. Most Influential Institutions and Countries

The institution with the highest number of documents was The Institut National de la Santé et de la Recherche Médicale (INSERM) (n = 36); it was followed by the institutions with the most citations related to this topic: Tongji Medical College (n = 24) and Huazhong University of Science and Technology (n = 24) (Table 4).

**Figure 4:**
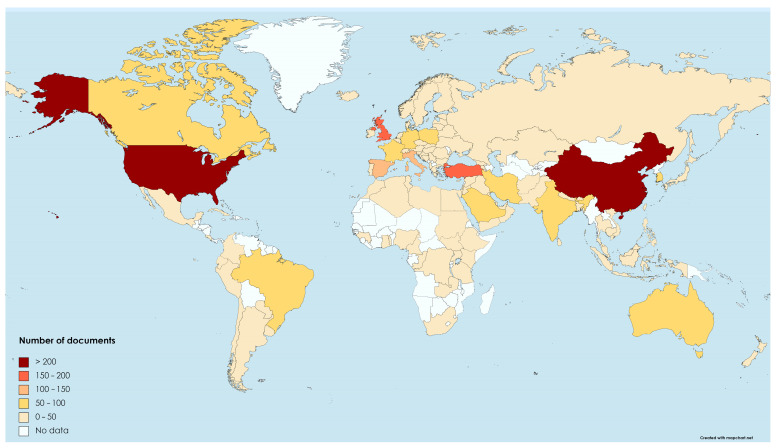
Worldwide distribution of publications on literature assessing fear of COVID-19 (2020–2022).

Overall, there were publications of literature assessing fear of COVID-19 from 112 different countries. Figure 4 depicts the global distribution of the contributing countries. Thus, China (n = 255) and the United States (n = 231) produced the vast majority of publications, while the United Kingdom (n = 172) and Turkey (n = 165) produced between 150 and 200. Italy and Spain produced between 100 and 150 publications, while 106 countries (94.64%) produced 100 or fewer documents.

### 3.6. Keywords

**Figure 5:**
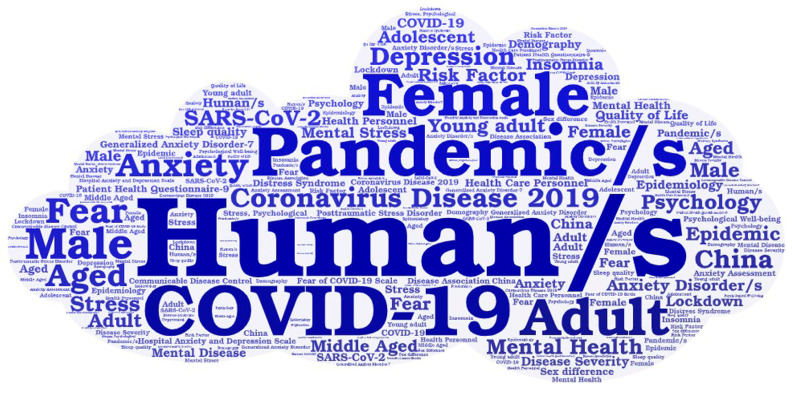
Tag crowd for keywords related to literature assessing fear of COVID-19 (2020–2022).

It should be noted that the keywords most commonly utilized were “human/s” (11.51%), “pandemic/s” (7.78%), “COVID-19” (6.41%), “female” (5.80%), “Coronavirus Disease 2019” (5.65%), “adult” (5.39%), “anxiety” (5.37%), and “male” (5.34%) (Figure 5).

## 4. Discussion

Our results show the growth in the annual citations in the documents from the literature assessing fear of COVID-19 that were published worldwide between 2020 and 2022. This rapid increase reflects an increasing concern about outbreak-related mental health [38]. In this context, citations are a valuable asset in academia because the number of citations is the most commonly used measurement to assess the quality of papers, journals, researchers, and universities [39]. According to our findings, journal articles are the most commonly published document type; these findings are in agreement with those of other studies [38,40]. This document type is commonly used to improve the development of specialized expertise in a particular study area [41]. English is the most widely used language in the research on fear of COVID-19 because it is the widely accepted language of communication in science around the world [42].

Although the pandemic has had a negative impact on many aspects of people’s lives [43], the majority of documents on fear of COVID-19 have centered on the area “Medicine”, surpassing other subject categories, which have received relatively limited scientific attention [40]. Of course, research priorities must be in line with the current global needs imposed by the COVID-19 pandemic [44]. Furthermore, it is crucial to acknowledge that governments and policymakers often prioritize waiting for conclusive scientific research rather than acting on the available evidence; this causes a delay that can hinder effective responses to the coronavirus and contribute to fear about the outbreak [45]. For instance, Jennings et al. [46] found predictors of indecision about the COVID-19 vaccine that were similar to predictors of indecision about older vaccines, such as lack of trust in government and the healthcare system and exposure to unregulated information about the COVID-19 vaccine through social media. However, we should pay special attention to the audience of policymakers, who must make quick evidence-based decisions [47].

The most basic measurement in a bibliometric analysis is the count of an author’s publications [48]. Here, the most productive author was from Nottingham Trent University (United Kingdom), which is also in the top four of the most influential institutions, with the UK being in the top three of the most productive countries publishing literature assessing fear of COVID-19 (2020–2022). The population of the United Kingdom reported particularly high levels of fear and anxiety about COVID-19 during the first national lockdown, possibly due to the country being one of the hardest hit [49]. Nevertheless, the vast majority of publications were produced by China and the United States (almost 20% of the total number of publications worldwide). However, no American author or institution had a large number of publications concentrated on this thematic area; the most prolific were M.S. Asghar, ranking 73rd among the authors, and Harvard Medical School, ranking 31st among the institutions. Even so, China and the United States have played a crucial role in mental health research because China was the main research force, especially in the early stage of the COVID-19 pandemic, which was first reported in Wuhan [38], and because of the overall strength of American academic research and its economy; meanwhile, in terms of total psychiatry publications, the United States has the most [50]. In addition, our results were in concordance with the great academic output of both countries on issues related to neuroscience [51]. Interestingly, The Institut National de la Santé et de la Recherche Médicale (INSERM) from France was the most productive institution in terms of publishing literature assessing fear of COVID-19. Before the COVID-19 pandemic, France was one of the most vaccine-hesitant countries in the world [52], and hesitancy toward COVID-19 vaccination remained higher than in most of the neighboring countries throughout the period [53]. According to Gagneux-Brunon et al. [54], a very high level of fear of COVID-19 was associated with support for a COVID-19 vaccination mandate for the general population in France. As a result, researching attitudes toward vaccine mandates in such a context can help to highlight the variety of factors that influence the acceptability of coercive measures [55].

Bibliometrics, a statistical analysis of published material, is based on the use of various measures or indicators, the majority of which rely on citation analysis. For example, a high number in the authors’ co-citations network map indicates a closer link between the researchers and their wider audience and a greater interest in a research area [56,57]. According to their usage, the ISI Impact Factor [58] and the SCImago Journal Rank are the most relevant indicators [59,60]. These scores are calculated by various institutions or companies that have their own academic journal data sources, the most important of which are: Journal Citation Report (JCR) [61] and SCImago Journal and Country Rank (SJR) [62]. In this context, the top 10 most active journals related to published literature assessing fear of COVID-19 were in Q1 and Q2 of both the Journal Citation Reports and the SCImago Journal Rankings. A journal’s impact factor is used as a proxy for the quality and expected impact of each paper published in it [63].

Finally, the analysis of keywords in a discipline can reveal research directions and hotspots [64]. In our study, the most frequently used keywords were “human/s”, “pandemic/s”, “COVID-19”, “female”, “Coronavirus Disease 2019”, “adult”, “anxiety”, and “male”. Apart from the keyword “human/s”, this study discovered that the research subjects in these types of studies were female, male, and adult, as they were in other bibliometric studies related to COVID-19 [65,66]. The other words, such as “pandemic/s”, “COVID-19”, “Coronavirus Disease 2019”, or “anxiety” indicated the dominance of keywords related to this theme, which is consistent with the results of other bibliometric studies about the COVID-19 pandemic [38,67,68].

Our study has some limitations. First, although Scopus is one of the largest databases, some journals have not been indexed; therefore, publications in these journals might have been ignored. In fact, there may be studies in this area that have been published in other databases. In future research, this bibliometric analysis should be replicated using specific databases, such as those in psychology or sociology, to provide a more comprehensive understanding of this topic. Moreover, it should be noted that this study only focused on the topic related to the published literature assessing fear of COVID-19. Finally, the total number of publications and citations was only correct at the time of the search. Despite all these limitations, this study presents various strengths. This article is among the first to analyze the detailed bibliometric indicators of the published literature assessing fear of COVID-19. Another strength of this study was that it used a search strategy with numerous terms.

Overall, our study presents not only a general overview of the published literature related to fear of COVID-19, but also a reference regarding the quality of the research into fear of pandemics and crisis management conducted by different authors, institutions, and countries concerning COVID-19. Bibliometric analyses over a specific time interval can serve as a framework for future comparisons between institutions and countries. Indeed, they reflect the distribution of research efforts among the different countries and even among the institutions within the same country [69]. Additionally, the present study can offer managers and researchers vital help in the possible decision-making situations in the future. In addition, we recommend repeating our study in future years when the pandemic is over to illustrate the extent to which the discussion on fear of COVID-19 has spread around the world. To conclude, the extensive scientific literature on the impact that the worldwide spread of COVID-19 has had on people’s quality of life has been analyzed through bibliometric analysis, providing new insights into patterns at the macroscopic and microscopic levels in the scholarly record.

## 5. Conclusions

A rapid growth in annual citations on the assessment of fear of COVID-19 followed the emergence of this disease, which indicates an increasing concern about this topic area. This bibliometric analysis provides an insight into the quality of the research into fear of pandemics and crisis management by diverse authors, institutions, and countries.

## Figures and Tables

**Table 1:** Subject categories focusing on published literature assessing fear of COVID-19 (2020–2022).

Subject Area	Frequencies (n)	Percentages (%)
Medicine	1502	84.91%
Psychology	203	11.48%
Neuroscience	182	10.29%
Environmental science	150	8.48%
Nursing	96	5.43%
Multidisciplinary	74	4.18%
Biochemistry, Genetics, and Molecular Biology	56	3.17%
Immunology and Microbiology	46	2.60%
Pharmacology, Toxicology, and Pharmaceutics	42	2.37%
Social science	41	2.32%

**Table 2:** Top 10 most productive journals related to published literature assessing fear of COVID-19 (2020–2022).

Journals	Categories	Number of Documents	Percentages	Citations	CPP ^1^	Quartile JCR ^a^	2022 Journal Impact Factor (IF) JCR ^a^	Quartile SJR ^†^	2022 Journal Impact Factor (IF) SJR ^†^
*International Journal of Environmental Research and Public Health*	Environmental Sciences/Public, Environmental, and Occupational Health	146	8.25%	2509	17.18	-	-	Q2	0.83
*Frontiers in Psychiatry*	Psychiatry	115	6.50%	1477	12.84	Q2	4.7	Q1	1.22
*PLoS One*	Multidisciplinary Sciences	62	3.50%	2520	40.65	Q2	3.7	Q1	0.89
*Journal of Affective Disorders*	Clinical Neurology/Psychiatry	46	2.60%	1175	25.54	Q1	6.6	Q1	1.99
*Frontiers in Public Health*	Public, Environmental, and Occupational Health	37	2.09%	170	4.59	Q1	5.2	Q1	1.13
*BMJ Open*	Medicine, General and Internal	29	1.64%	220	7.59	Q2	2.9	Q1	1.06
*Journal of Clinical Medicine*	Medicine, General and Internal	28	1.58%	248	8.86	Q2	3.9	Q1	0.94
*Psychiatry Research*	Psychiatry	27	1.53%	4303	159.37	Q1	11.3	Q1	2.14
*Frontiers in Psychology*	Psychology, Multidisciplinary	25	1.41%	193	7.72	Q1	3.8	Q2	0.89
*BMC Psychiatry*	Psychiatry	24	1.36%	300	12.50	Q2	4.4	Q1	1.29

^1^ CPP: Citations per publication; ^a^ JCR: Journal Citation Report; ^†^ SJR: SCImago Journal and Country Rank.

**Table 3:** Top 10 most productive authors related to published literature assessing fear of COVID-19 (2020–2022).

Rank	Author	Country	Institution	Number of Publications	First Author Position	Last Author Position	Other Author Position	Single Author	Number of Citations	CPP ^1^	h-Index (Period 2020–2022)
1	M. D. Griffiths	United Kingdom	Nottingham Trent University	21	0	8	13	0	744	35.43	15
2	S. Chung	South Korea	University of Ulsan College of Medicine	19	1	14	4	0	147	7.74	8
3	A. H. A. Pakpour	Iran	Qazvin University of Medical Sciences	18	2	10	6	0	563	31.28	11
4	C. Y. Ling	Taiwan	National Cheng Kung University College of Medicine	16	2	2	12	0	561	35.06	11
5	T. Cheung	Hong Kong	Hong Kong Polytechnic University	10	0	0	10	0	100	10.00	6
6	C. Fernández de las Peñas	Spain	Universidad Rey Juan Carlos	10	10	0	0	0	76	7.60	5
7	C. Pieh	Austria	University for Continuing Education Krems	10	5	5	0	0	766	76.60	7
8	A. M. Rogowska	Poland	Uniwersytet Opolski	10	4	0	6	0	160	16.00	6
9	Y.-T. Xiang	Macau	University of Macau	10	0	10	0	0	95	9.50	5
10	V. Hernández Barrera	Spain	Universidad Rey Juan Carlos	9	0	1	8	0	124	13.78	7

^1^ CPP: Citations per publication.

**Table 4:** Top 10 most productive and influential institutions publishing literature assessing fear of COVID-19 (2020–2022).

Rank	Institutions	Countries	Number of Documents	Citations
1	Institut National de la Santé et de la Recherche Médicale (INSERM)	France	36	556
2	Tongji Medical College	China	24	1087
3	Huazhong University of Science and Technology	China	24	926
4	Nottingham Trent University	United Kingdom	23	787
5	University of Health Sciences	Turkey	23	140
6	Hong Kong Polytechnic University	Hong Kong	22	508
7	The University of Hong Kong	Hong Kong	21	698
8	King’s College London	United Kingdom	21	280
9	King Saud University	Saudi Arabia	21	397
10	Universidad Rey Juan Carlos	Spain	21	271

## Data Availability

The data presented in this study are available as Supplementary Material (File S1: Research data).

## Supplementary Materials

The following supporting information can be downloaded at: https://www.mdpi.com/article/10.3390/clinpract14030054/s1, Table S1: Search strategy for published literature assessing fear of COVID-19, File S1: Research data.
