# Supplementary material for: A Worldwide Bibliometric Analysis of Published Literature Assessing Fear of COVID-19
Source: Clin Pract. 2024 Apr 23;14(3):672–84. doi: 10.3390/clinpract14030054 (PMC11130842; doi:10.3390/clinpract14030054)
Supplement: Supplementary file 1 [file clinpract-14-00054-s001.zip › Supplementary file/Supplementary Materials.docx]

Supplementary Materials

A worldwide bibliometric analysis of published literature assessing fear of COVID-19

Jesús Cebrino^1^ and Silvia Portero de la Cruz^2,3^*

^1^ Department of Preventive Medicine and Public Health, Faculty of Medicine, University of Seville, Avda. Doctor Fedriani, S/N, 41009 Seville, Spain; email: [jcebrino@us.es](mailto:jcebrino@us.es) (J.C.).

^2^ Department of Nursing, Pharmacology and Physiotherapy, Faculty of Medicine and Nursing, University of Córdoba, Avda. Menéndez Pidal, S/N, 14071 Córdoba, Spain; e-mail: [n92pocrs@uco.es](mailto:n92pocrs@uco.es) (S.P.d.l.C.).

^3^ Research Group GE10 Clinical and Epidemiological Research in Primary Care, Instituto Maimónides de Investigación Biomédica de Córdoba (IMIBIC), Hospital Universitario Reina Sofía, 14071 Córdoba, Spain; e-mail: [n92pocrs@uco.es](mailto:n92pocrs@uco.es) (S.P.d.l.C.)

***** Correspondence: [n92pocrs@uco.es](mailto:n92pocrs@uco.es); Tel.: +34-957-218-106

**Table S1.** Search strategy for published literature assessing fear of COVID-19.

| Scopus database (17/01/2023) | |
| --- | --- |
| Concept | **Search strategy** |
| (a) Fear | TITLE-ABS-KEY(“fear*” OR “scare” OR “terror” OR “dread” OR “fright*” OR “afraid” OR “panic*” OR “avoid*” OR “phobi*” OR “phobi* disorder*” OR “phobi* neuros*” OR “panic disorder*” OR “concern*” OR “anxi*” OR “anticipatory anxi*” OR “anxi* disorder*” OR “anxi* neuros*” OR “anxi* management” OR “worr*” OR “perceived danger*” OR “perceived risk*” OR “perceived safety” OR “psychological safety” OR “sense of safety”) |
| (b) COVID-19 | TITLE-ABS-KEY(“COVID 2019*” OR “COVID19 virus” OR “COVID-19 virus” OR “COVID19*” OR “COVID 19*” OR “covid”) AND TITLE-ABS-KEY(“Wuhan coronavirus” OR “Wuhan seafood market pneumonia virus”) OR TITLE-ABS-KEY(“coronavirus 2019” OR “corona virus 2019” OR “coronavirus disease” OR “coronavirus disease 2019 virus” OR “coronavirus disease 2019” OR “coronavirus disease-19” OR “2019 novel coronavirus diseases”) OR TITLE-ABS-KEY(“SARS-CoV-2” OR “SARS2” OR “SARS-CoV” OR “Severe acute respiratory syndrome coronavirus 2” OR “SARS-CoV-2019” OR “SARS-CoV-19” OR “SARS coronavirus 2”) OR TITLE-ABS-KEY(“2019-nCoV” OR “2019 novel coronavirus” OR “2019-novel CoV” OR “2019 novel coronavirus infection” OR “novel coronavirus” OR “novel coronavirus 2019” OR “novel coronavirus pneumonia” OR “nCoV” OR “nCoV2019” OR “nCoV-2019”) OR TITLE-ABS-KEY(“2019-nCoV disease” OR “2019 novel coronavirus disease” OR “2019-nCoV infection”) |
| (c) Instruments assessing fear of COVID-19 | TITLE-ABS-KEY(“Fear of COVID-19 Scale” OR “FCV-19S” OR “Fear Perception and Magnitude of the Issue” OR “MED-COVID-19” OR “Scale of COVID-19 related psychological distress in healthy public” OR “CORPD” OR “COVID-19 Phobia Scale” OR “C19P-S” OR “Hospital Anxiety and Depression Scale” OR “HADS” OR “Severity Measure for Specific Phobia-Adult” OR “SMSP-A” OR “General Anxiety Disorder” OR “GAD-7” OR “Perceived Vulnerability to Disease” OR “PVD”) |
